# Supplementary material for: Metabolic Alteration Analysis of Steroid Hormones in Niemann–Pick Disease Type C Model Cell Using Liquid Chromatography/Tandem Mass Spectrometry
Source: Int J Mol Sci. 2022 Apr 18;23(8):4459. doi: 10.3390/ijms23084459 (PMC9025463; doi:10.3390/ijms23084459)
Supplement: Supplementary file 1 [file ijms-23-04459-s001.zip › Fig_S_2.5.pptx]

## Slide 1
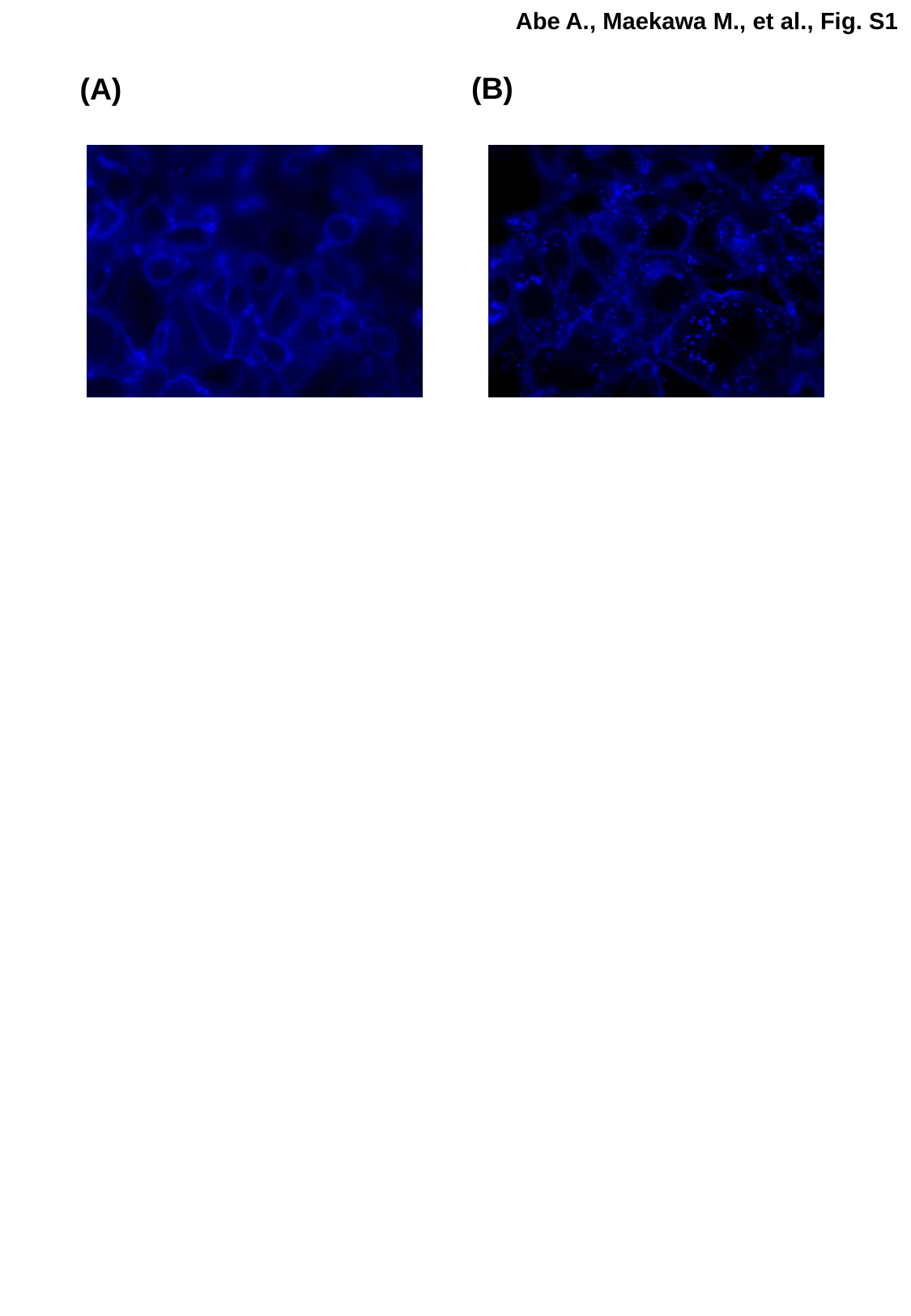

Abe A., Maekawa M., et al., Fig. S1
(B)
(A)

## Slide 2
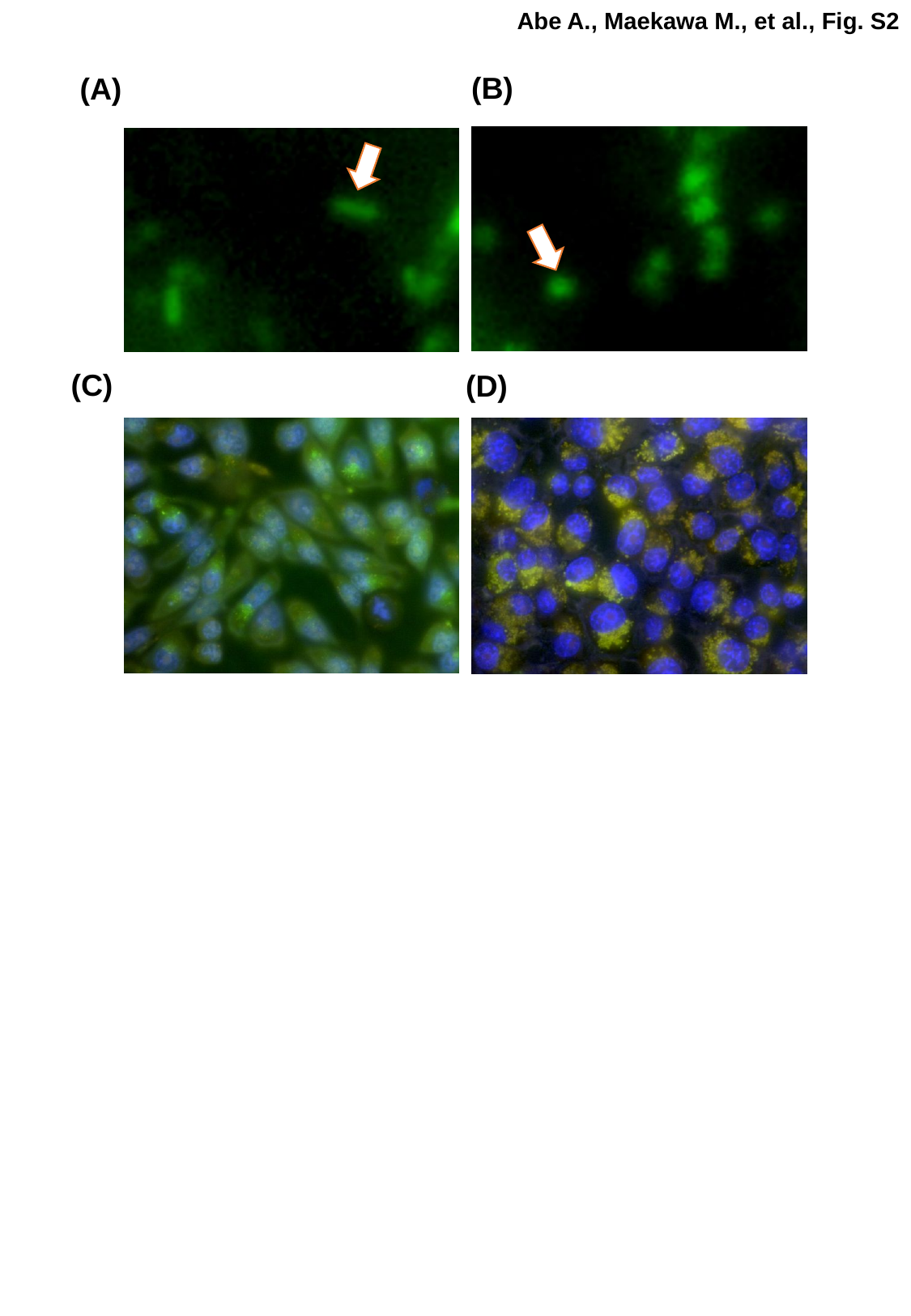

Abe A., Maekawa M., et al., Fig. S2
(B)
(A)
(C)
(D)

## Slide 3
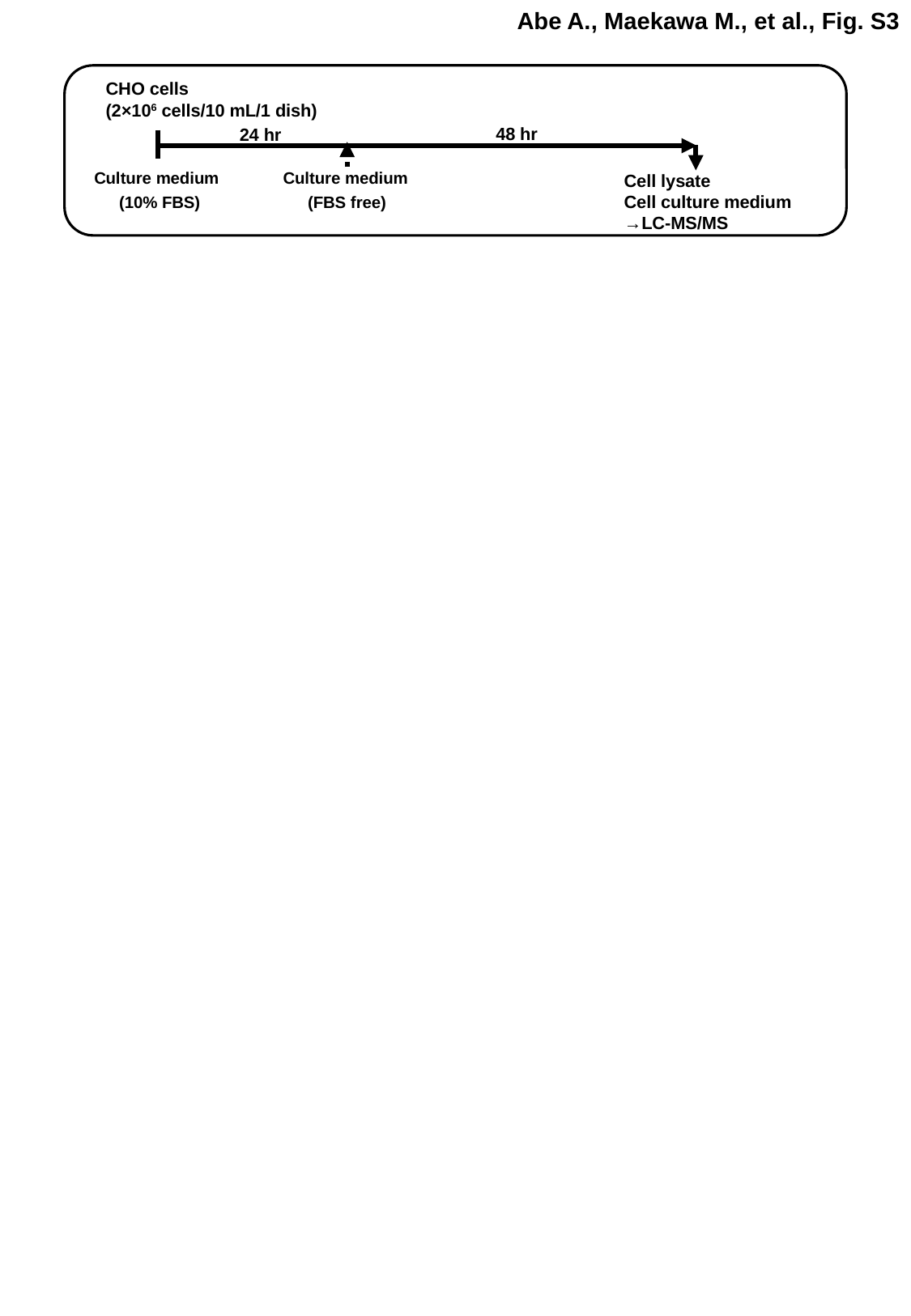

Abe A., Maekawa M., et al., Fig. S3
CHO cells
(2×10^6^ cells/10 mL/1 dish)
48 hr
24 hr
Culture medium
Culture medium
Cell lysate
Cell culture medium
→LC-MS/MS
(10% FBS)
(FBS free)
3
