# Supplementary material for: Metabolic Alteration Analysis of Steroid Hormones in Niemann–Pick Disease Type C Model Cell Using Liquid Chromatography/Tandem Mass Spectrometry
Source: Int J Mol Sci. 2022 Apr 18;23(8):4459. doi: 10.3390/ijms23084459 (PMC9025463; doi:10.3390/ijms23084459)
Supplement: Supplementary file 1 [file ijms-23-04459-s001.zip › Table S1_2.5.pdf]

Supplementary Table S1. Absolute recovery rate with each preparation condition of liquid-liquid extraction.

| Analytes | Absolute recovery rate (%) | | | | |
|---|---|---|---|---|---|
| Solvent 1 | AcOEt | tBME | AcOEt | tBME | AcOEt/tBME (1:1, v/v) |
| Solvent 2 | AcOEt | tBME | tBME | AcOEt | AcOEt/tBME (1:1, v/v) |
| Testosterone | 97.8 | 99.0 | 100 | 99.0 | 102 |
| Androsterone | 93.4 | 99.6 | 95.5 | 100 | 105 |
| Epiandrosterone | 98.1 | 110 | 107 | 107 | 117 |
| Cortisol | 99.6 | 101 | 104 | 104 | 105 |
| Cortisone | 96.7 | 95.6 | 95.2 | 92.7 | 97.1 |
| Corticosterone | 92.9 | 86.9 | 88.6 | 85.1 | 89.5 |
| Aldosterone | 104 | 120 | 116 | 125 | 122 |
| Pregnenolone | 93.8 | 106 | 108 | 101 | 108 |
| Progesterone | 99.3 | 101 | 101 | 102 | 104 |
| Estrone | 93.7 | 91.8 | 100 | 100 | 95.2 |
| Estradiol | 99.5 | 102 | 102 | 101 | 103 |
| Estriol | 97.4 | 96.7 | 97.9 | 96.7 | 101 |

AcOEt, ethyl acetate; LLE, liquid–liquid extraction; tBME, tert-butyl methyl ether.
