# Supplementary material for: Metabolic Alteration Analysis of Steroid Hormones in Niemann–Pick Disease Type C Model Cell Using Liquid Chromatography/Tandem Mass Spectrometry
Source: Int J Mol Sci. 2022 Apr 18;23(8):4459. doi: 10.3390/ijms23084459 (PMC9025463; doi:10.3390/ijms23084459)
Supplement: Supplementary file 1 [file ijms-23-04459-s001.zip › Table S2_2.5.pdf]

Supplementary Table S2. Matrix factor of each analytes and ISs.

(A) Matrix factor of each analytes and ISs.

Analytes	Cell-cultured medium	Cell suspension
Testosterone	116	109
Androsterone	90.8	105
Epiandrosterone	72.4	109
DHEA	134	98.3
Cortisol	107	101
Cortisone	113	98.0
Corticosterone	96.2	105
Aldosterone	112	82.8
Pregnenolone	88.4	96.4
Progesterone	124	115
Estrone	101	73.1
Estradiol	92.8	70.3
Estriol	101	75.9
Testosterone-$^{2}H_3$	117	113
Androsterone-$^{2}H_4$	84.9	97.0
DHEA-$^{2}H_6$	130	101
Cortisol-$^{13}C_3$	104	99.9
Aldosterone-$^{2}H_7$	106	93.2
Pregnenolone-$^{13}C_2$,$^{2}H_2$	81.0	103
Progesterone-$^{2}H_9$	128	121
Estrone-$^{2}H_4$	97.7	72.4
Estradiol-$^{13}C_3$	89.1	71.1
Estriol-$^{13}C_3$	97.7	75.6

IS, internal standard.

(B) IS normalized MF of each analytes.

		IS normalized MF (%)	
Analytes	IS	Cell cultured medium	Cell suspension
Testosterone	Testosterone-$^{2}H_3$	99.0	96.4
Androsterone	Androsterone-$^{2}H_4$	107	108
Epiandrosterone	Androsterone-$^{2}H_4$	85.3	113
DHEA	DHEA-$^{2}H_6$	104	97.1
Cortisol	Cortisol-$^{13}C_3$	104	101
Cortisone	Cortisol-$^{13}C_3$	109	98.1
Corticosterone	Cortisol-$^{13}C_3$	92.9	105
Aldosterone	Aldosterone-$^{2}H_7$	112	88.8
Pregnenolone	Pregnenolone-$^{13}C_2$,$^{2}H_2$	109	93.3
Progesterone	Progesterone-$^{2}H_9$	96.7	95.6
Estrone	Estrone-$^{2}H_4$	104	101
Estradiol	Estradiol-$^{13}C_3$	104	99.0
Estriol	Estriol-$^{13}C_3$	103	101

MF, matrix factor.
