# Supplementary material for: Metabolic Alteration Analysis of Steroid Hormones in Niemann–Pick Disease Type C Model Cell Using Liquid Chromatography/Tandem Mass Spectrometry
Source: Int J Mol Sci. 2022 Apr 18;23(8):4459. doi: 10.3390/ijms23084459 (PMC9025463; doi:10.3390/ijms23084459)
Supplement: Supplementary file 1 [file ijms-23-04459-s001.zip › Table S3_2.5.pdf]

Supplementary Table S3. Calibration curves.

Analytes	IS	Calibration range (ng/mL)	Equation regression	*R*
Testosterone	Testosterone-$^{2}H_3$	0.01-100	y = 1.09 x + 0.00270	0.995
Androsterone	Androsterone-$^{2}H_4$	0.03-30	y = 0.500 x + 0.00000557	0.995
Epiandrosterone	Androsterone-$^{2}H_4$	0.1-100	y = 1.06 x + 0.00609	0.999
DHEA	DHEA-$^{2}H_6$	0.03-100	y = 252 x + 1.58	0.992
Cortisol	Cortisol-$^{13}C_3$	0.001-100	y = 0.881 x + 0.000285	0.995
Cortisone	Cortisol-$^{13}C_3$	0.001-100	y = 1.68 x + 0.000304	0.995
Corticosterone	Cortisol-$^{13}C_3$	0.01-30	y = 1.36 x + 0.00116	0.992
Aldosterone	Aldosterone-$^{2}H_7$	0.03-10	y = 0.546 x - 0.000125	0.994
Pregnenolone	Pregnenolone-$^{13}C_2$,$^{2}H_2$	0.03-100	y = 0.191 x + 0.000253	1.000
Progesterone	Progesterone-$^{2}H_9$	0.003-100	y = 0.675 x + 0.000151	0.999
Estrone	Estrone-$^{2}H_4$	0.001-100	y = 1.00 x + 0.000149	0.999
Estradiol	Estradiol-$^{13}C_3$	0.003-100	y = 0.862 x + 0.000123	0.992
Estriol	Estriol-$^{13}C_3$	0.001-100	y = 0.942 x + 0.000138	0.999

R, correlation coefficient.
