# Supplementary material for: Metabolic Alteration Analysis of Steroid Hormones in Niemann–Pick Disease Type C Model Cell Using Liquid Chromatography/Tandem Mass Spectrometry
Source: Int J Mol Sci. 2022 Apr 18;23(8):4459. doi: 10.3390/ijms23084459 (PMC9025463; doi:10.3390/ijms23084459)
Supplement: Supplementary file 1 [file ijms-23-04459-s001.zip › Table S4_2.5.pdf]

Supplementary Table S4. Intra-day assay and inter-day assay in cell samples

(A) Intra-day assay for compounds with analysis of positive ion mode

| | Precision (CV, %) | | | Accuracy (RE, %) | | |
|---|---|---|---|---|---|---|
| Analytes | QCL 0.6 ng/mL | QCM 6 ng/mL | QCH 50 ng/mL | QCL 0.6 ng/mL | QCM 6 ng/mL | QCH 50 ng/mL |
| Testosterone | 2.34 | 2.37 | 2.41 | 10.6 | 6.82 | -10.4 |
| Androsterone | 10.6 | 7.06 | 5.42 | 2.34 | 4.00 | −5.66 |
| Epiandrosterone | 1.24 | 8.99 | 5.23 | 2.22 | 3.50 | −7.58 |
| DHEA | 8.97 | 7.12 | 5.34 | −9.25 | −2.13 | −6.82 |
| Cortisol | 1.81 | 5.23 | 4.39 | 10.3 | 6.88 | −8.36 |
| Cortisone | 0.932 | 6.76 | 1.04 | 12.1 | 10.5 | −10.0 |
| Corticosterone | 0.770 | 5.32 | 2.75 | 8.91 | −1.24 | −17.5 |
| Aldosterone | 3.68 | 7.73 | 1.43 | −30.0 | −12.3 | −8.14 |
| Pregnenolone | 6.88 | 6.07 | 8.10 | −1.32 | −6.67 | −9.43 |
| Progesterone | 1.60 | 3.85 | 3.68 | 4.59 | 0.759 | −8.90 |

(B) Intra−day assay for compounds with analysis of negative ion mode

| | Precision (CV, %) | | | Accuracy (RE, %) | | |
|---|---|---|---|---|---|---|
| Analytes | QCL 0.06 ng/mL | QCM 0.6 ng/mL | QCH 50 ng/mL | QCL 0.06 ng/mL | QCM 0.6 ng/mL | QCH 50 ng/mL |
| Estrone | 4.72 | 5.47 | 3.34 | 9.43 | 5.22 | 2.25 |
| Estradiol | 4.06 | 1.99 | 3.03 | 0.140 | 4.89 | −4.71 |
| Estriol | 11.6 | 2.71 | 5.70 | −3.78 | −4.58 | −18.5 |

CV, coefficient of variation; N.Q., not quantified; QCH, high concentration of quality control level; QCL, low concentration of quality control level; QCM, medium concentration of quality control level; RE, Relative error.

(C) Inter−day assay for compounds with analysis of positive ion mode

| Analytes | Precision (CV, %) QCL 0.6 ng/mL | Precision (CV, %) QCM 6 ng/mL | Precision (CV, %) QCH 50 ng/mL | Accuracy (RE, %) QCL 0.6 ng/mL | Accuracy (RE, %) QCM 6 ng/mL | Accuracy (RE, %) QCH 50 ng/mL |
|---|---|---|---|---|---|---|
| Testosterone | 9.91 | 4.00 | 2.51 | −15.0 | 3.27 | −9.03 |
| Androsterone | 24.2 | 7.78 | 5.87 | −5.73 | 0.425 | −3.05 |
| Epiandrosterone | 23.9 | 6.83 | 7.46 | −16.3 | 1.86 | −3.83 |
| DHEA | 13.0 | 9.50 | 3.49 | −3.14 | −1.969 | −3.26 |
| Cortisol | 3.29 | 2.60 | 3.45 | 1.42 | 0.563 | −4.41 |
| Cortisone | 3.57 | 1.70 | 2.78 | −2.51 | 0.415 | −11.1 |
| Corticosterone | 6.95 | 3.31 | 2.22 | −2.50 | 0.271 | −8.36 |
| Aldosterone | 16.0 | 6.38 | 7.04 | −5.72 | 18.0 | 10.7 |
| Pregnenolone | 11.0 | 8.23 | 6.55 | 0.848 | −4.48 | −1.70 |
| Progesterone | 6.49 | 2.82 | 2.48 | −2.57 | −0.582 | −3.95 |

(D) Inter−day assay for compounds with analysis of negative ion mode

| Analytes | Precision (CV, %) QCL 0.06 ng/mL | Precision (CV, %) QCM 0.6 ng/mL | Precision (CV, %) QCH 50 ng/mL | Accuracy (RE, %) QCL 0.06 ng/mL | Accuracy (RE, %) QCM 0.6 ng/mL | Accuracy (RE, %) QCH 50 ng/mL |
|---|---|---|---|---|---|---|
| Estrone | 6.31 | 4.18 | 3.32 | −1.90 | −7.49 | −8.59 |
| Estradiol | 5.49 | 4.42 | 3.39 | 5.44 | 8.04 | −9.25 |
| Estriol | 5.46 | 5.11 | 2.59 | 5.28 | −0.281 | −10.8 |

CV, coefficient of variation; N.Q., not quantified; QCH, high concentration of quality control level; QCL, low concentration of quality control level; QCM, medium concentration of quality control level; RE, Relative error.
