# Supplementary material for: Metabolic Alteration Analysis of Steroid Hormones in Niemann–Pick Disease Type C Model Cell Using Liquid Chromatography/Tandem Mass Spectrometry
Source: Int J Mol Sci. 2022 Apr 18;23(8):4459. doi: 10.3390/ijms23084459 (PMC9025463; doi:10.3390/ijms23084459)
Supplement: Supplementary file 1 [file ijms-23-04459-s001.zip › Table S5_2.5.pdf]

Supplementary Table S5. Intra-day assay and inter-day assay in medium samples.

(A) Intra-day assay for compounds with analysis of positive ion mode

Analytes	Precision (CV, %)			Accuracy (RE, %)		
	QCL 0.6 ng/mL	QCM 6 ng/mL	QCH 50 ng/mL	QCL 0.6 ng/mL	QCM 6 ng/mL	QCH 50 ng/mL
Testosterone	2.01	1.44	1.98	−5.95	6.50	−6.25
Androsterone	9.57	1.64	9.21	−4.11	1.54	−1.16
Epiandrosterone	11.5	1.82	3.78	−35.6	−3.88	−2.15
DHEA	15.0	9.50	5.01	10.6	9.52	3.48
Cortisol	3.48	1.48	2.47	0.853	3.80	−1.44
Cortisone	5.20	2.12	2.27	−0.080	2.92	−7.66
Corticosterone	2.81	1.81	1.64	−6.27	−0.319	−6.76
Aldosterone	17.8	2.77	4.94	−4.77	2.97	9.99
Pregnenolone	5.13	7.70	6.70	−11.1	2.73	7.83
Progesterone	5.65	3.98	1.79	3.71	2.76	−1.65

(B) Intra−day assay for compounds with analysis of negative ion mode

Analytes	Precision (CV, %)			Accuracy (RE, %)		
	QCL 0.06 ng/mL	QCM 0.6 ng/mL	QCH 50 ng/mL	QCL 0.06 ng/mL	QCM 0.6 ng/mL	QCH 50 ng/mL
Estrone	1.54	4.96	0.969	1.92	3.73	−0.69
Estradiol	3.09	2.56	6.27	−4.00	2.45	−10.1
Estriol	7.57	8.01	0.347	5.87	2.82	−6.76

CV, coefficient of variation; N.Q., not quantified; QCH, high concentration of quality control level; QCL, low concentration of quality control level; QCM, medium concentration of quality control level; RE, Relative error.

(C) Inter−day assay for compounds with analysis of positive ion mode

Analytes	Precision (CV, %) QCL 0.6 ng/mL	Precision (CV, %) QCM 6 ng/mL	Precision (CV, %) QCH 50 ng/mL	Accuracy (RE, %) QCL 0.6 ng/mL	Accuracy (RE, %) QCM 6 ng/mL	Accuracy (RE, %) QCH 50 ng/mL
Testosterone	9.69	5.17	1.33	−15.5	2.20	−6.92
Androsterone	17.4	3.89	7.48	1.45	2.02	−0.563
Epiandrosterone	11.9	4.92	3.84	−12.9	0.414	−0.862
DHEA	14.4	6.96	4.18	4.98	3.87	1.07
Cortisol	2.89	3.46	3.13	1.98	2.83	−0.326
Cortisone	3.59	2.91	4.44	1.62	2.97	−4.49
Corticosterone	5.18	3.84	3.46	−2.53	−1.02	−6.44
Aldosterone	20.3	9.28	8.85	−13.0	−6.24	1.06
Pregnenolone	9.14	6.80	8.14	−1.91	2.24	2.53
Progesterone	6.60	4.98	1.98	−0.492	−0.605	−1.35

(D) Inter−day assay for compounds with analysis of negative ion mode

Analytes	Precision (CV, %) QCL 0.06 ng/mL	Precision (CV, %) QCM 0.6 ng/mL	Precision (CV, %) QCH 50 ng/mL	Accuracy (RE, %) QCL 0.06 ng/mL	Accuracy (RE, %) QCM 0.6 ng/mL	Accuracy (RE, %) QCH 50 ng/mL
Estrone	11.8	4.82	2.25	−3.50	−0.198	−0.0681
Estradiol	11.4	3.13	3.69	−5.58	2.25	−9.80
Estriol	11.1	5.75	2.61	0.0550	0.348	−8.52

CV, coefficient of variation; N.Q., not quantified; QCH, high concentration of quality control level; QCL, low concentration of quality control level; QCM, medium concentration of quality control level; RE, Relative error.
