# Supplementary material for: Metabolic Alteration Analysis of Steroid Hormones in Niemann–Pick Disease Type C Model Cell Using Liquid Chromatography/Tandem Mass Spectrometry
Source: Int J Mol Sci. 2022 Apr 18;23(8):4459. doi: 10.3390/ijms23084459 (PMC9025463; doi:10.3390/ijms23084459)
Supplement: Supplementary file 1 [file ijms-23-04459-s001.zip › Table S6_2.5.pdf]

Supplementary Table S6. Amounts of steroid hormones in cell of wild-type cells and NPC model cells.

	Wild-type cells (pg/10^6cells)			NPC model cells (pg/10^6cells)		
Testosterone *	166	±	2.80	5.30	±	0.117
Androsterone *	5.45	±	0.317	0.265	±	0.459
Epiandrosterone	6.12	±	6.03	18.4	±	24.8
DHEA	4.16	±	0.886	7.44	±	4.09
Cortisol	0.00333	±	0.00578	0.0544	±	0.473
Cortisone	0.0872	±	0.0167	0.0633	±	0.0229
Corticosterone	6.21	±	3.86	11.7	±	9.85
Aldosterone	0.408	±	0.0606	0.342	±	0.148
Pregnenolone	2.86	±	0.968	2.79	±	2.78
Progesterone *	4.27	±	0.0426	2.87	±	0.180
Estrone *	0.166	±	0.00918	0.0483	±	0.0120
Estradiol	0.204	±	0.0351	0.246	±	0.104
Estriol	N.Q.			0.0472	±	0.0231

Each data represents the mean ± S.D. (Control, n = 3). N.Q., not quantified. * means that the significantly difference were shown in $P<0.05$. The significantly difference was investigated with Wilcoxon's test.

WT, wild-type CHO cell; NPC, *Npc1* gene trap CHO cell.
