# Supplementary material for: Metabolic Alteration Analysis of Steroid Hormones in Niemann–Pick Disease Type C Model Cell Using Liquid Chromatography/Tandem Mass Spectrometry
Source: Int J Mol Sci. 2022 Apr 18;23(8):4459. doi: 10.3390/ijms23084459 (PMC9025463; doi:10.3390/ijms23084459)
Supplement: Supplementary file 1 [file ijms-23-04459-s001.zip › Table S7_2.5.pdf]

Supplementary Table S7. Amounts of steroid hormones in cell culturing medium of wild-type cells and NPC model cells.

	Wild type cell ($pg/10^6$cells)			NPC model cell ($pg/10^6$cells)		
Testosterone	26.4	±	0.685	25.3	±	0.685
Androsterone		N.Q.			N.Q.	
Epiandrosterone	−252	±	200	−121	±	168
DHEA	38.4	±	27.2	24.1	±	48.6
Cortisol	1.58	±	0.811	0.788	±	0.197
Cortisone *	1.75	±	1.11	0.219	±	0.317
Corticosterone	−118	±	61.1	−77.4	±	74.0
Aldosterone	2.47	±	1.05	3.37	±	1.80
Pregnenolone		N.Q.			N.Q.	
Progesterone *	17.1	±	1.18	11.2	±	0.997
Estrone *	1.34	±	0.269	0.542	±	0.244
Estradiol *	3.31	±	0.953	1.15	±	0.562
Estriol		N.Q.			N.Q.	

Each data represents the mean ± S.D. (Control, n = 3). N.Q., not quantified. * means that the significantly difference were shown in $P<0.05$. The significantly difference was investigated with Wilcoxon's test.

WT, wild−type CHO cell; NPC, *Npc1* gene trap CHO cell.
