# Supplementary material for: Metabolic Alteration Analysis of Steroid Hormones in Niemann–Pick Disease Type C Model Cell Using Liquid Chromatography/Tandem Mass Spectrometry
Source: Int J Mol Sci. 2022 Apr 18;23(8):4459. doi: 10.3390/ijms23084459 (PMC9025463; doi:10.3390/ijms23084459)
Supplement: Supplementary file 1 [file ijms-23-04459-s001.zip › Table S8_2.5.pdf]

Supplementary Table S8. Ion source parameters.

Polarity	CUR (psi)	CAD (unit)	IS (V)	TEM (°C)	GS1 (psi)	GS2 (psi)
Positive ion mode	15	12	5500	500	70	60
Negative ion mode	25	10	-4500	550	60	60

CAD, collision gas; CUR, curtain gas; GS, ion source gas; IS, ionspray voltage; TEM, temperature.
